# The Association Between Unrecognized Hypovolemia and Head-Up Tilt Testing Positivity

**DOI:** 10.1016/j.jacadv.2025.102460

**Published:** 2025-12-09

**Authors:** Owais S. Mian, David M. Newman, Yash Shrestha, Russell J. de Souza, Sheldon M. Singh

**Affiliations:** a Division of Cardiology, Kingston Health Sciences, Queen’s University, Kingston, Ontario, Canada; b Schulich Heart Program, Sunnybrook Health Sciences Centre, Temerty Faculty of Medicine, University of Toronto, Toronto, Ontario, Canada; c Department of Health Research Methods, Evidence, and Impact, Faculty of Health Sciences, McMaster University, Hamilton, Ontario, Canada; d Population Health Research Institute, Hamilton, Ontario, Canada; e Heersink School of Global Health and Social Medicine, McMaster University, Hamilton, Ontario, Canada

**Keywords:** Bayesian probability statistics, head-up tilt table testing, syncope

Vasovagal syncope is complex. Ventricular mechanoreceptor activation due to ventricular under filling and subsequent sympathetic withdrawal and vagal tone enhancement results in vasodilation and/or bradycardia which decreases cerebral perfusion. Head-up tilt-table testing (HUTT) is a noninvasive test that recreates these conditions to confirm this diagnosis. The diagnostic utility of HUTT is debated because its low sensitivity (between 20% and 75%) results in a high number of false negative test results.^1^

**What is the clinical question being addressed?**

Is hypovolemia determined with postural vital signs associated with a positive tilt-test result?

**What is the main finding?**

Hypovolemia was associated with less nitrate use and a positive tilt-test result.

Standard HUTT protocols mandate fasting prior to HUTT. Fasting may result in hypovolemia priming the physiologic cascade thereby reducing the false negative test rate. Postural vital signs are a pragmatic, objective, and validated marker of hypovolemia.^2^ In this study, we assessed the association between abnormal postural vital signs at the time of HUTT and HUTT positivity in patients with suspected vasovagal syncope.

## Methods

Sunnybrook Health Sciences Centre is a large regional cardiac center in Ontario, Canada. This retrospective cohort study included all patients aged ≥18 years who underwent HUTT between December 1, 2014, and May 1, 2024. The study protocol was approved by the Sunnybrook Research Ethics Board. Given its retrospective design and use of deidentified data, the requirement for patient consent was waived.

Patients were instructed to fast for 6 hours prior to HUTT. A positive HUTT was defined as the development of syncope or altered consciousness accompanied by a fall in heart rate and/or blood pressure.

As highlighted in the *Journal of the American Medical Association*’s Rational Clinical Exam series,^2^ postural vital signs are a valuable clinical tool to confirm a diagnosis of hypovolemia. A postural increase in heart rate >30 beats/min or a drop in systolic blood pressure >20 mm Hg at 2 minutes of standing have a likelihood ratio of 1.7 and 1.5, respectively, for diagnosing hypovolemia.^3^ Patients with a sustained elevation in heart rate without a drop in blood pressure persisting at 10 minutes were excluded from this analysis and classified as having postural orthostatic tachycardia syndrome (POTS). Baseline heart rate and blood pressure were determined in a supine position prior to tilting. These values were measured at the 2-minute mark of tilting. Patients with either an increase in heart rate >30 beats/min or drop in systolic blood pressure >20 mm Hg at 2 minutes of tilting compared to when supine were classified as hypovolemic.

Demographic and clinical differences between groups with and without hypovolemia were compared using chi-squared tests. A multivariable logistic regression model was fitted to identify predictors of a positive HUTT. Variables included in the model were selected for clinical relevance and included age (per 10 years), sex (female reference), referring physician (cardiologist reference), and the presence of hypovolemia at 2 minutes of testing. Morning vs afternoon test time was included in the model to assess for an association between test positivity and overnight fasting which may be longer in duration. Season testing was performed was also included to assess whether insensible fluid loss in the summer was associated with a positive test. The multivariable ORs of a positive test, with 95% CIs and *P* values are reported.

Statistical analyses were performed using SAS software, version 9.4 (SAS Institute).

## Results

Of 1,063 patients undergoing HUTT at our institution during the study period, 205 were excluded due to a test indication of POTS or actual evidence of POTS on HUTT. The final study cohort consisted of 858 patients with suspected vasovagal syncope. The mean age of this cohort was 48.8 years, 61% were female, and 87.8% were referred by a cardiologist. Hypovolemia was present in 20.6% of the cohort. HUTT was positive in 41.9% of the cohort and more common in patients who were hypovolemic (54.6% vs 39.1%; *P* = 0.0003). Nitroglycerine was less frequently used in patients with hypovolemia compared to those without hypovolemia (51.8% vs 62.1%; *P* = 0.015). Table 1 summarizes the result of the multivariate logistic regression analysis. The presence of markers of hypovolemia was associated with the greatest odds of a positive HUTT (OR: 1.88; 95% CI: 1.34-2.65; *P* = 0.0003).

**Table 1:** Predictors of Positive Tilt Table Test in Those Referred for Vasovagal Syncope

	OR	95% CI	*P* Value
Age (continuous variable, per 10 y)	0.98	0.92-1.06	0.66
Sex (female vs male)	1.09	0.82-1.45	0.56
Referring physician (cardiologist vs noncardiologist)	1.53	0.99-2.37	0.054
Presence of hypovolemia	1.88	1.34-2.65	0.0003
Season			0.13
Winter	Ref		
Spring	1.26	0.85-1.89	0.81
Summer	1.15	0.77-1.72	0.60
Autumn	1.56	1.07-2.28	0.04
Test time of day (afternoon vs morning)	0.98	0.73-1.32	0.89

## Discussion

Hypovolemia at the time of HUTT was associated with almost two-fold increased odds of a positive test result. The findings highlight the importance of adequate fasting to promote hypovolemia prior to HUTT in patients with syncope.

Previous echocardiographic studies have demonstrated that individuals with vasovagal syncope exhibit exaggerated reductions in left ventricular end-diastolic volume during orthostatic stress compared with healthy controls.^3^ This decreased volume may activate mechanoreceptors within the ventricular wall resulting in bradycardia and vasodilation which can lead to fainting. Our results suggest that hypovolemia may enhance this reflex thereby increasing HUTT positivity.

Patients who were hypovolemic at the time of HUTT less frequently required nitrates. This finding is important due to concerns of a reduction in test specificity with the administration of nitrates during HUTT. Furthermore, this pragmatic approach to identify hypovolemia based on data already available during a tilt test may provide valuable information when interpreting a negative test. A negative test with hypovolemia may reflect a true negative test. Thus, achieving adequate hypovolemia prior to HUTT may improve Bayesian probability statistics associated with interpreting the results of HUTT.

Consistently achieving hypovolemia in patients undergoing HUTT may be difficult to achieve. Indeed, with the provision of uniform fasting recommendations, this was achieved in one in five patients in our cohort. This is remarkable, as our local fasting requirement of 6 hours is longer than the 2 to 4 hours recommended in the European Society of Cardiology guidelines.^4^ Our findings should, however, stress the importance of providing firm instruction on fasting to patients and consideration of longer durations of fasting prior to HUTT.

Limitations to our work include its single-center nature. Additionally, we lacked biochemical or echocardiographic markers to corroborate hypovolemia. While some may consider this a limitation, change in postural vitals is validated to diagnose hypovolemia, and unlike laboratory testing or echocardiography which require additional testing, time and cost, postural vitals are readily available during the HUTT. Finally, it is possible patients with primary autonomic dysfunction or orthostatic hypotension may have been misclassified as primary hypovolemia.

In conclusion, the presence of hypovolemia determined by changes in postural vital signs during HUTT is associated with a positive HUTT result. We strongly suggest clinicians performing HUTT be attentive of changes in postural vitals within the first 2 minutes of a HUTT and incorporate this when interpreting the results of a negative HUTT. We recommend further work to optimize fasting protocols to ensure hypovolemia is achieved when performing HUTT.

## Funding support and author disclosures

This work was supported by a generous donation from the Horgan Family and Sunnybrook Hospital Foundation. The authors have reported that they have no relationships relevant to the contents of this paper to disclose.
